# Regenerative Therapy as an Adjunct to Periapical Surgery: A Case Report

**DOI:** 10.5005/jp-journals-10005-1139

**Published:** 2012-02-24

**Authors:** Vivek Chaturvedy, Shefali Chaturvedy

**Affiliations:** Professor and Head, Department of Periodontics, Rajasthan Dental College and Hospital, Jaipur, Rajasthan, India, e-mail: vivek_chaturvedi@yahoo.com; Senior Lecturer, Department of Pedodontics and Preventive Dentistry Rajasthan Dental College and Hospital, Jaipur, Rajasthan, India

**Keywords:** Radicular cyst, Apicoectomy, Bone graft

## Abstract

Large periapical defects may adversely affect the success rate of endodontic surgery. Use of regenerative therapy may enhance the prognosis of such teeth. A case of traumatized upper anterior teeth with infected radicular cyst and associated sinus tract reported to the dental hospital. A periradicular surgical procedure was performed to remove the nonhealing pathological tissue. To augment the repair a bioactive bone graft material was placed. Six months interim results showed positive outcome of application of graft.

**How to cite this article:** Chaturvedy V, Chaturvedy S. Regenerative Therapy as an Adjunct to Periapical Surgery: A Case Report. Int J Clin Pediatr Dent 2012;5(1):75-77.

## INTRODUCTION

Inspite of advancements in the field of dentistry, treatment of endodontically involved teeth with large periapical defect still remains a daunting task for a dental surgeon. Associated disruption of the cortical plate and existence of dentoalveolar sinus tract results in poor prognosis of the involved tooth.^1^

In such cases, repair process is dictated not only by a hermetically sealed retrograde obturation, but also the hard tissue dimensions of the remaining marginal and periapical bone tissue.^2^ Delay or alterations in healing have been reported when lesion size is greater than 5 mm.^3^

The concept of regenerative therapy entails utilization of periosteal grafts with the potential to stimulate bone formation. It allows cellular regrowth of defects caused by pathosis or surgical trauma and has resulted in development of grafts, membrane or barriers that are used in periodontal therapy.^4^ Likewise, different studies have demonstrated that this technique can also be successfully applied in endodontic surgery as these materials act as reservoir and matrix for deposition of new bone.^5^

## CASE REPORT

**Fig. 1:**
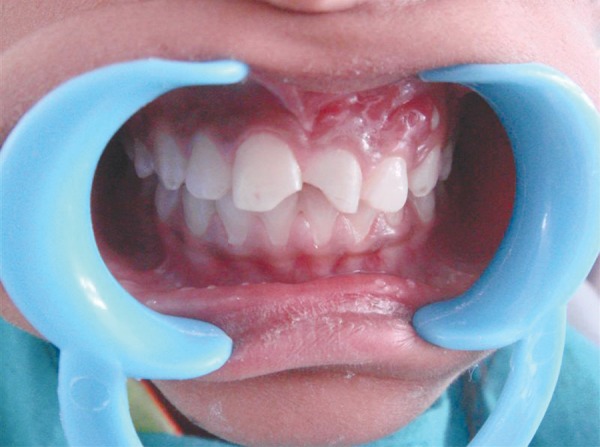
Preoperative

A 12-year-old female patient reported to the Department of Pedodontics and Preventive Dentistry, Rajasthan Dental College and Hospital with the complaint of discolored upper left anterior tooth with recurrent discharge of pus in relation to the same tooth since the last 4 to 5 months. History revealed that she had a fall 2 years back resulting in fracture

of upper front teeth. Intraoral examination showed 11 with Ellis class II fracture and 21 with Ellis class III fracture and discoloration. An infected sinus was present in relation to 21 with pus discharge (Fig. 1). There was no pain on percussion and no tenderness in the periapical region. Grade II mobility was present in relation to 21, whereas 22 showed grade I mobility. A nonvital response was elicited from 21 and 22 on using thermal and electric pulp tester.

**Fig. 2:**
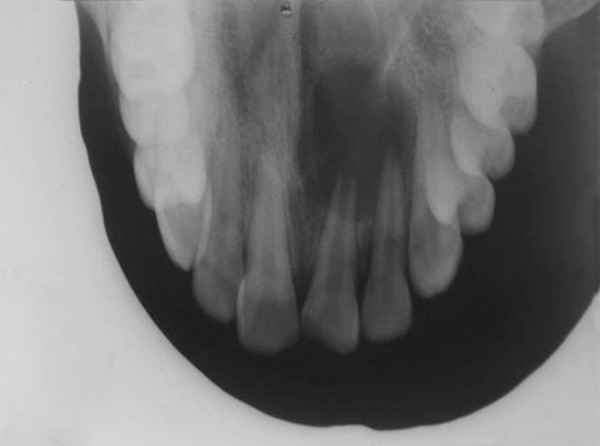
Preoperative diagnostic X-ray

Maxillary occlusal view revealed incomplete root formation with 21 and an oval shaped periapical radioluscency about 3 × 2.5 cm with clear-cut borders involving the periapical area and apical one-third of roots of 21 and 22 (Fig. 2). The case was diagnosed to be infected radicular cyst involving 21 and 22. Root canal treatment was initiated in 21 and 22, meanwhile periapical surgery was planned as there was constant pus discharge.

**Fig. 3:**
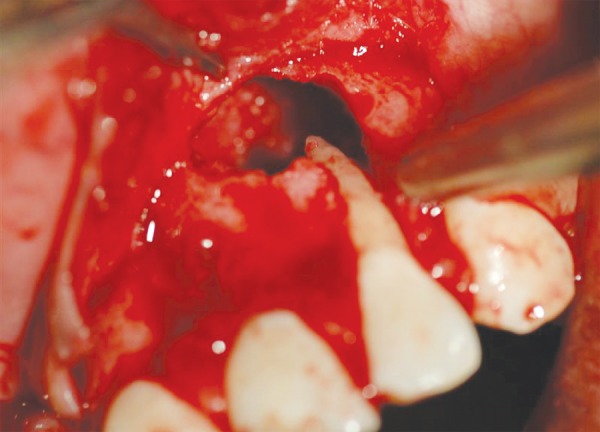
Bone defect

Routine blood investigations and oral prophylaxis were carried out. Patient was prescribed prophylactic antibiotic a day prior to surgery. A trapezoidal mucoperiosteal flap was reflected using crevicular incisions with limiting vertical incision on distal aspect of 11 and 23 under local anesthesia. Complete loss of buccal cortical plate was observed with 21 (Fig. 3). Complete curettage of the defect including cystic lining was achieved. After thorough curettage of the granulomatous tissue, the area was thoroughly irrigated using betadine solution. The root canal was obturated with gutta- percha points under proper isolation. Retrograde filling was done using glass ionomer cement.

**Fig. 4:**
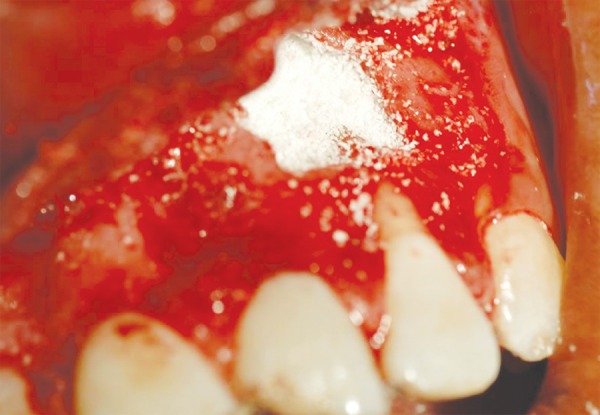
Defect with bone graft

Slight bleeding was induced from the bony cavity and PerioGlas^®^ (Nova bone) ceramic crystals were directly compacted into the bony defect (Fig. 4). The mucoperiosteal flap was approximated with interrupted sutures. Periopack was applied on the surgical area.

**Fig. 5:**
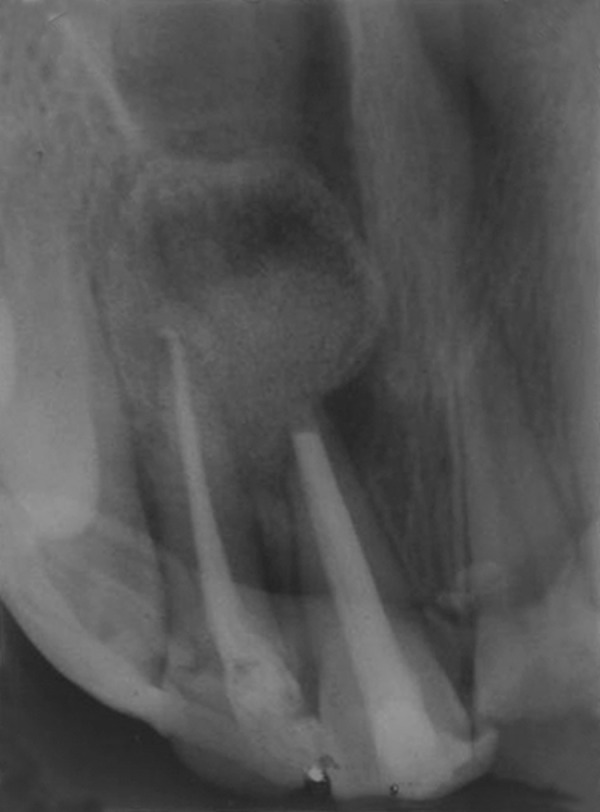
Postoperative X-ray

**Fig. 6:**
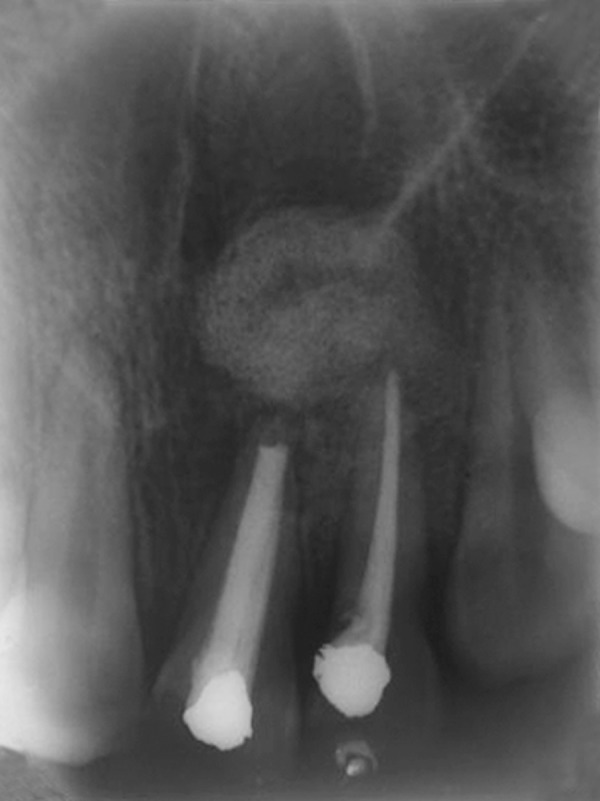
Three months postoperative X-ray

**Fig. 7:**
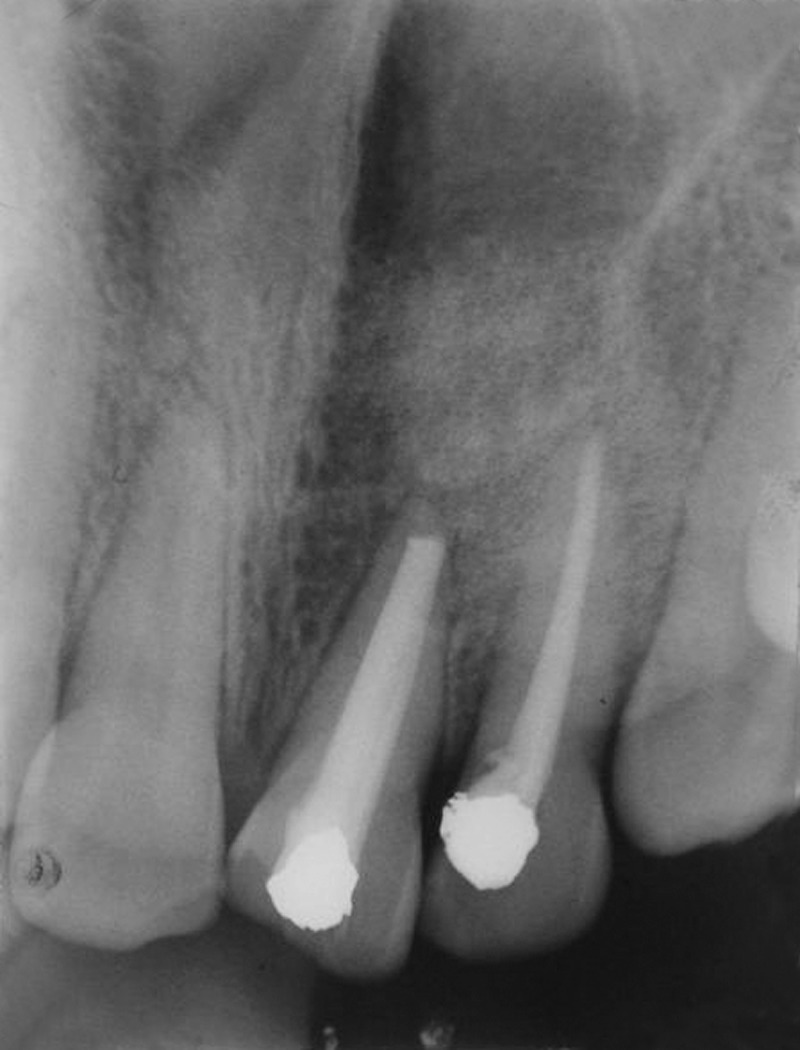
Six months postoperative X-ray

Immediate postoperative radiograph revealed satisfactory bone fill in the defect area (Fig. 5). Sutures were removed by the end of first week and there was uneventful soft tissue healing. Clinically, by end of first month there was absence of pus discharge and mobility was reduced to grade I in 21 and was absent in 22. At the end of third month mobility in both teeth had completely disappeared. Subsequently, IOPAs were taken at 1 week, 1, 3 and 6 months interval which demonstrated increasing levels of radiopacity signifying successful uptake of graft and positive bone regeneration. One week postoperative radiograph showed increased radiopacity of the material within the bony defect. Radiograph taken in the 3rd month showed a diffuse radiopaque area and increased calcification surrounding the graft (Fig. 6). Radiopacity with foci of pattern progressing from ground glass to spiculed appearance was observed indicating healing calcifying defect at the end of 6 months (Fig. 7). 11 and 23 were tested for vitality using thermal and electric test and both the teeth tested vital during succeeding visits.

## DISCUSSION

Bone grafts can be used to achieve favorable healing and regeneration of the periapical defect area after degranulation.^6^ In endodontic surgical sites these materials are employed with the intention to act as bone fills and scaffolds which facilitate wound healing, normal trabecular bone formation and prevent proliferation of the oral epithelium into such defects ensuring healthy clinical outcome.^7^

Bone grafts are rarely indicated in pediatric cases, as the high turnover rate of cells and rich blood supply warrants quick healing. However, treatment of nonvital young permanent teeth with large open apices, extensive periapical radioluscencies and thin dentinal walls warrant special deliberation.^8^ Retrograde restoration with glass ionomer cement was preferred over apexification due to infection and absence of healthy surrounding periapical bone. It also provided immediate and predictable results as opposed to apexification procedure.

PerioGlas^®^ is a bioactive glass composed primarily of silica, calcium, sodium and phosphorous. It is an amorphous, crystalline and completely absorbable material. Its principle mode of action is by osteostimulation which stimulates and accelerates new bone formation in an osseous defect. In addition the osteoconductive effect leads to new bone formation at the defect margin which penetrates to center of the graft. Adjunctive benefits include antimicrobial, anti- inflammatory and hemostatic effect. These are a result of alkaline nature of cations released by the graft which ensures rapid healing. PerioGlas^®^ has shown greater new cementum and alveolar bone formation than other materials.^9^

**Fig. 8:**
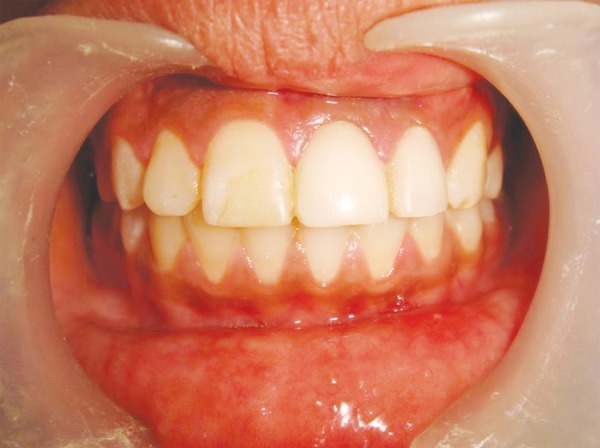
Postoperative with composite build up and acrylic crown

Assessment of success or failure after periradicular surgery is based on clinical and radiographic criteria. Post surgery patient showed uneventful healing and improvement in the status of tooth supporting structures (Fig. 8). The positive effect of bone graft was clearly observed in succeeding radiographs. Results were in accordance to guidelines given by Wood NK, Goaz PW.^10^

The present case thus highlights the positive effect of bone grafting when used in conjugation with endodontic surgical procedure. It showed good clinical and radiographic signs of healing and may contribute to successful treatment of such cases. However, further clinical trials could address the utilization of these materials vis-a-vis pediatric dentistry.
